# Injecting drugs alone during an overdose crisis in Vancouver, Canada

**DOI:** 10.1186/s12954-022-00701-w

**Published:** 2022-11-17

**Authors:** Alexa Norton, Kanna Hayashi, Cheyenne Johnson, JinCheol Choi, M-J Milloy, Thomas Kerr

**Affiliations:** 1 British Columbia Centre on Substance Use, St. Paul’s Hospital, 400–1045 Howe Street, Vancouver, BC V6Z 2A9 Canada; 2 Division of Social Medicine, Department of Medicine, University of British Columbia, Vancouver, Canada; 3 School of Nursing, University of British Columbia, Vancouver, Canada; 4 Faculty of Health Sciences, Simon Fraser University, Burnaby, Canada

**Keywords:** Injecting alone, Solitary injection, Using alone, People who inject drugs, Injection drug use, Overdose, Overdose prevention

## Abstract

**Background:**

Settings throughout Canada and the USA continue to experience crises of overdose death due to the toxic unregulated drug supply. Injecting drugs alone limits the potential for intervention and has accounted for a significant proportion of overdose deaths, yet the practice remains understudied. We sought to examine the practice of injecting alone among people who inject drugs (PWID) in Vancouver, Canada.

**Methods:**

Data were derived from two prospective cohorts of people who use drugs between June 2016 and November 2018. This analysis was restricted to participants who, in the previous 6 months, reported any injection drug use. Rates of injecting alone were categorized as always, usually, sometimes, or occasionally. We fit a multivariable generalized linear mixed model to identify factors associated with injecting drugs alone.

**Results:**

Among 1070 PWID who contributed 3307 observations, 931 (87%) reported injecting alone at least once during the study period. In total, there were 729 (22%) reports of always injecting alone, 722 (21.8%) usually, 471 (14.2%) sometimes, 513 (15.5%) occasionally, and 872 (26.4%) never. In a multivariable model, factors positively associated with injecting drugs alone included male sex (adjusted odds ratio [AOR] 1.69; 95% confidence interval [CI] 1.20–2.37), residence in the Downtown Eastside neighbourhood (AOR 1.43; 95% CI 1.08–1.91), binge drug use (AOR 1.36; 95% CI 1.08–1.72), and experiencing physical or sexual violence or both (AOR 1.43; 95% CI 1.00–2.03). Protective factors included Indigenous ancestry (AOR 0.71; 95% CI 0.52–0.98) and being in a relationship (AOR 0.30; 95% CI 0.23–0.39).

**Conclusion:**

We observed that injecting alone, a key risk for overdose mortality, was common among PWID in Vancouver. Our findings underline the need for additional overdose prevention measures that are gender-specific, culturally appropriate, violence- and trauma-informed, and available to those who inject alone.

## Introduction

Many settings in Canada and the USA are contending with an overdose crisis driven largely by the proliferation of synthetic opioids, especially fentanyl, in the drug supply. This is true of British Columbia (BC), Canada, where a public health emergency was declared in April 2016 due to increasing overdose deaths. In response, interventions such as naloxone, overdose prevention/supervised consumption sites (OPS/SCS), and opioid agonist therapy (OAT) were expanded, with additional interventions such as the prescription of pharmaceutical alternatives to unregulated drugs (or “prescribed safer supply”) emerging more recently [28]. Despite these interventions, the number of overdose deaths rose from 993 in 2016 to 1562 in 2018, a 57% increase [5]. With the emergence of the novel coronavirus pandemic in 2020, overdose deaths rose to 2265 in 2021, a 128% increase from 2016 [5], indicating a continued need for novel responses to the overdose crisis.

Previous research suggests that many people who inject drugs (PWID) inject alone and do so for several reasons: to avoid sharing drugs and injection equipment; for privacy, convenience, and comfort; to reduce the potential for assault, robbery, and intimate partner violence; as a result of a preference to inject at home; as a result of frequent disruptions in social networks; and shame and stigma surrounding drug use [1, 17, 30, 33, 39, 46]. Despite a high concentration of harm reduction services in Vancouver, BC, including those in supportive housing settings [20, 33, 34] and accessible virtually [27], a majority of unregulated overdose deaths take place among people who use drugs (PWUD) alone and indoors, including in private market and supportive housing, shelters, single-room occupancy (SROs) and other hotels [5], [29]. “Alone” is defined as being unaccompanied at the time of consumption, although given the complexity of toxic drug overdose, it can be difficult to collect reliable data on the circumstances surrounding deaths from using alone [29]. In 2021, 84% of toxic drug deaths in BC occurred indoors [5]. This makes responding to overdoses challenging and, in some instances, impossible. Since the emergence of the overdose crisis in North America, few cohort studies have examined the phenomenon of injecting alone [37], and we know of none undertaken in environments with a range of conventional and innovative overdose prevention programs (e.g. housing-based, OPS/SCS, a range of opioid agonist therapies, prescribed safer supply). Amid rising overdose death rates, there is a need to better understand the practice of injecting alone and inform targeted overdose prevention measures by identifying the various characteristics associated with this practice. Leveraging data from cohorts of PWUD in Vancouver, we sought to examine potential drivers of injecting alone and further characterize this particular overdose risk behaviour.

## Methods

Two ongoing prospective cohort studies of people who use unregulated drugs in Vancouver, Canada, were the source of data for this study: the Vancouver Injection Drug Users Study (VIDUS) and the AIDS Care Cohort to Evaluate exposure to Survival Services (ACCESS). Both have been described elsewhere [26, 47]. VIDUS enrols HIV-negative adults who report injecting an unregulated drug in the month prior to enrolment, and ACCESS is a cohort of adults living with HIV who report using an unregulated drug in the month prior to enrolment. Both cohorts have been recruited through snowball sampling and street outreach since 1996. At baseline and semi-annually, participants complete an interviewer-administered questionnaire that gathers sociodemographic data and information about drug use patterns. Study instruments and follow-up procedures are harmonized to enable combined analyses. Written informed consent is obtained from participants who receive a $40 (CAN) honorarium for each study visit. The University of British Columbia/Providence Health Care Research Ethics Board provided ethical approval for both studies.

Data for the present study were collected between June 2016 and November 2018, after the public health emergency was declared and using the most recent data available at the time of study. We restricted our analysis to participants who reported any injection drug use in the previous 6 months. Our primary outcome of interest was injecting drugs alone in the previous 6 months at any period during follow-up, defined by answering “How often do you inject alone with nobody around?” Participants responded on a Likert scale: always (100% of the time), usually (75% or more), sometimes (74% to 26%), occasionally (25% or less), or never (0%). Although higher frequency injecting alone may involve different behavioural drivers and pose escalating risk, in the context of an increasingly toxic drug supply (fentanyl was detected in 85% of overdose deaths in 2018 [6]), we determined that injecting alone at any frequency poses significant overdose risk and created a dichotomous variable (yes vs. no) where responses were coded affirmatively if participants responded always, usually, sometimes, or occasionally, and no if they responded never. Based on a review of the relevant literature and our longstanding experience in the local environment, a range of other explanatory variables hypothesized to be associated with injecting alone were included, such as demographic, health, cultural, and social factors. We considered Indigenous ancestry (self-identified vs. other) given evidence indicating that PWUD of Indigenous ancestry experience a disproportionate burden of overdose and other drug-related harms [12, 25]. Other time-fixed characteristics considered included: age (per year older), sex (male vs. female); sexual orientation, defined as lesbian, gay, bisexual, Two-Spirit, or other vs. straight; relationship status, defined as legally married, common law, or regular partner vs. other; depression symptomology, measured using the Center for Epidemiological Studies Depression Scale (CES-D) (≥ 22 vs. < 22) at baseline; and posttraumatic stress disorder (PTSD), measured one time using the PTSD Checklist for *DSM*-5 (PCL-5) (≥ 31 vs. < 31) [16]. Characteristics measured in the previous 6 months at each study interview that we considered included: employment, defined as income generation from a regular or temporary job or self-employment (yes vs. no); homelessness (yes vs. no), defined as being without any shelter; Downtown Eastside (DTES) residence (yes vs. no), a neighbourhood in Vancouver that is disproportionately affected by overdose deaths [44]; incarceration (yes vs. no); sex work, defined as exchanging sex for money, gifts, food, shelter, clothes, etc. (yes vs. no); drug dealing (yes vs. no); at least weekly OPS/SCS use (yes vs. no); non-fatal overdose (yes vs. no); and experiencing violence, defined as physical or sexual or both (yes vs. no). Drug (injection and non-injection) and alcohol use behaviours measured in the previous 6 months included: daily heroin (yes vs. no), daily fentanyl (yes vs. no), daily cocaine (yes vs. no), daily crack cocaine (yes vs. no), daily methamphetamine (yes vs. no), any binge drug use (yes vs. no), and alcohol binging (yes vs. no). Binge use was defined as periods when drugs or alcohol were used more than usual.

Since repeated measures were available, we applied a generalized linear mixed model (GLMM) with logit-link function that considers only random intercepts for simplicity. Bivariate GLMM analysis was used to determine factors associated with injecting alone in the previous 6 months. We then fit a multivariable GLMM that included variables significant at the level of *p* < 0.10 in bivariate analyses. A sub-analysis was conducted using a different cut-off (always vs. usually, sometimes, and occasionally) to determine factors associated with the highest frequency of injecting alone. All *p* values are two-sided, and analyses were conducted using R software version 4.0.5 (R Foundation for Statistical Computing, Vienna, Austria).

## Results

Analyses were restricted to 1070 individuals who contributed 3307 observations in total, with a median number of three per participant (interquartile range [IQR] 2–5). Among participants there was a median age of 46.3 years (IQR 36.3–53.8), 421 (39.4%) females, and 422 (39.4%) people who self-identified as Indigenous (Table 1). Of the sample, 931 (87%) reported injecting drugs alone in the previous 6 months at least once during the study period. In total, there were 729 (22%) reports of always injecting alone, 722 (21.8%) usually, 471 (14.2%) sometimes, 513 (15.5%) occasionally, and 872 (26.4%) never.

**Table 1:** Baseline characteristics of 1070 people who use drugs, stratified by injecting alone, Vancouver, Canada, 2016–2018

Characteristic	Total *N* (%) (*n* = 1070)	Injecting alone (%)		*p* value
		Yes (*n* = 931)	No (*n* = 139)	
Age (median, IQR)	46 (36–54)	46 (37–54)	45 (35–53)	0.151^d^
Male sex (vs. female)	649 (60.7)	580 (62.3)	69 (49.6)	0.005
Indigenous ancestry (yes vs. no)	422 (39.4)	358 (38.5)	64 (46)	0.113
LGBT(yes vs. no)	184 (17.2)	149 (16)	35 (25.2)	0.011
Stable relationship (yes vs. no)	365 (34.1)	284 (31)	81 (58.3)	< 0.001
Employment^a^ (yes vs. no)	256 (23.9)	217 (23.3)	39 (28.1)	0.241
Homelessness (yes vs. no)	257 (24)	225 (24.2)	32 (23)	0.915
DTES residence^a^ (yes vs. no)	735 (68.7)	645 (69.3)	90 (64.8)	0.282
Recent incarceration^a^ (yes vs. no)	72 (6.7)	67 (7.2)	5 (3.6)	0.145
Sex work^a^ (yes vs. no)	155 (14.5)	133 (14.3)	22 (15.8)	0.605
Recent violence^a^ (yes vs. no)	182 (17)	158 (17)	24 (17.3)	0.904
Daily heroin use^a^ (yes vs. no)	381 (35.6)	330 (35.5)	51 (36.7)	0.776
Daily fentanyl use^a^ (yes vs. no)	25 (2.3)	23 (2.5)	2 (1.4)	0.761
Daily cocaine use^a^ (yes vs. no)	69 (6.5)	60 (6.4)	9 (6.5)	1
Daily crack cocaine use^a^ (yes vs. no)	113 (10.6)	98 (10.5)	15 (10.8)	0.883
Daily meth use^a^ (yes vs. no)	213 (19.9)	188 (20.2)	25 (18)	0.648
Binge drug use^a^ (yes vs. no)	490 (45.8)	437 (46.9)	53 (38.1)	0.055
Binge alcohol use^a^ (yes vs. no)	189 (17.7)	166 (17.8)	23 (16.6)	0.812
Drug dealing^a^ (yes vs. no)	272 (25.4)	237 (25.5)	35 (25.2)	1
Weekly OPS/SCS use^a^ (yes vs. no)	253 (23.6)	220 (23.6)	33 (23.7)	1
Non-fatal overdose^a^ (yes vs. no)	205 (19.2)	186 (20)	19 (13.7)	0.083
Depression^b^ (> = 22 at Baseline vs. < 22)	646 (60.4)	572 (61.4)	74 (53.2)	0.02
PTSD^c^ (≥ 31 vs. < 31)	303 (28.3)	279 (30)	24 (17.3)	0.013

All drug use variables refer to non-injection and injection use

*DTES* Downtown Eastside, *IQR* interquartile range

^a^Behaviours/events in the past 6 months

^b^Measured using the Centre for Epidemiologic Studies Depression Scale (CES-D) – >  = 22 at Baseline

^c^Measured using the Posttraumatic Stress Disorder Checklist for DSM-5 (PCL-5)-cut-point score of ≥ 31

^d^Wilcoxon’s rank-sum test

In multivariable analyses (shown in Table 2), male sex (adjusted odds ratio [AOR] 1.69; 95% confidence interval [CI] 1.20–2.37), DTES residence (AOR 1.43; 95% CI 1.08–1.91), binge drug use (AOR = 1.36; 95% CI: 1.08–1.72), and experiencing violence (AOR 1.43; 95% CI 1.00–2.03) remained independently associated with increased odds of injecting alone, while Indigenous ancestry (AOR 0.71; 95% CI 0.52–0.98) and being in a relationship (AOR 0.30; 95% CI 0.23–0.39) were associated with decreased odds.

**Table 2:** Bivariate and multivariable GLMM analyses of factors associated with injecting drugs alone among 3307 observations from people who inject drugs in Vancouver, Canada, 2016–2018

Variable	Unadjusted		Adjusted	
	Odds ratio (95% CI)	*p* value	Odds ratio (95% CI)	*p* value
*Age*				
(per year older)	1.01 (0.99–1.02)	0.234	–	–
*Sex*				
(male vs. female)	2.42 (1.75–3.35)	** < 0.001**	1.69 (1.20–2.37)	**0.002**
*Indigenous ancestry*				
(Self-identified vs. other)	0.55 (0.39–0.76)	** < 0.001**	0.71 (0.52–0.98)	**0.037**
*Sexual orientation*				
(LGBT vs. straight)	0.89 (0.61–1.30)	0.547	–	–
*Stable relationship*				
(yes vs. no)	0.27 (0.20–0.35)	** < 0.001**	0.30 (0.23–0.39)	** < 0.001**
*Employment*^*a*^				
(yes vs. no)	1.24 (0.92–1.66)	0.152	–	–
*Homelessness*				
(yes vs. no)	1.09 (0.80–1.51)	0.579	–	–
*DTES residence*^*a*^				
(yes vs. no)	1.37 (1.02–1.83)	**0.034**	1.43 (1.08–1.91)	**0.014**
*Recent incarceration*^*a*^				
(yes vs. no)	1.03 (0.63–1.69)	0.907	–	–
*Sex work*^*a*^				
(yes vs. no)	0.72 (0.49–1.06)	**0.092**	–	–
*Violence*^*a*^				
(yes vs. no)	1.38 (0.97–1.95)	**0.072**	1.43 (1.00–2.03)	**0.045**
*Daily heroin use*^*a*^				
(yes vs. no)	0.99 (0.76–1.29)	0.937	–	–
*Daily fentanyl use*^*a*^				
(yes vs. no)	1.45 (0.73–2.90)	0.293	–	–
*Daily cocaine use*^*a*^				
(yes vs. no)	0.93 (0.56–1.55)	0.781	–	–
*Daily crack cocaine use*^*a*^				
(yes vs. no)	1.19 (0.77–1.83)	0.430	–	–
*Daily meth use*^*a*^				
(yes vs. no)	1.13 (0.82–1.56)	0.445	–	–
*Binge drug use*^*a*^				
(yes vs. no)	1.35 (1.07–1.70)	**0.012**	1.36 (1.08–1.72)	**0.009**
*Binge alcohol use*^*a*^				
(yes vs. no)	0.99 (0.71–1.37)	0.946	–	–
*Drug dealing*^a^				
(yes vs. no)	1.03 (0.76–1.39)	0.845	–	–
*Weekly OPS/SCS use*^*a*^				
(yes vs. no)	0.97 (0.73–1.28)	0.811	–	–
*Non-fatal overdose*^*a*^				
(yes vs. no)	0.84 (0.62–1.12)	0.236	–	–
*Depression*^b^				
(≥ 22 vs. < 22)	1.32 (0.93–1.89)	0.124	–	–
*PTSD*^*c*^				
(≥ 31 vs. < 31)	1.28 (0.87–1.89)	0.217	–	–

All drug use variables refer to non-injection and injection use

*CI* confidence interval, *DTES* Downtown Eastside

^**a**^Behaviours/events in the past 6 months

^b^Measured using the Centre for Epidemiologic Studies Depression Scale (CES-D) – >  = 22 at Baseline

^c^Measured using the Posttraumatic Stress Disorder Checklist for DSM-5 (PCL-5)-cut-point score of ≥ 31

In a sub-analysis, age (AOR 1.02; 95% CI 1.01–1.04) and employment (AOR 1.45; 95% CI 1.10–1.91) were associated with increased odds of always injecting alone, while drug dealing (AOR 0.68; 95% CI 0.49–0.94), being in a relationship (AOR 0.30; 95% CI 0.23–0.39), and weekly OPS/SCS use (AOR 0.41; 95% CI 0.29–0.57) were associated with decreased odds.

## Discussion

Amid an ongoing overdose crisis, our study found that among 1070 people who inject drugs, 87% reported injecting alone in the previous 6 months at least once between June 2016 and November 2018. Among 3307 total observations, there were 729 reports (22%) of always injecting alone. Binge drug use was associated with higher odds of injecting alone, as were the demographic factors of male sex and residence in Vancouver’s Downtown Eastside. A unique finding was that those who experienced violence were more likely to inject alone. We further identified that being in a relationship and having Indigenous ancestry were protective factors against injecting alone. In a sub-analysis, older age and employment were associated with higher odds of always injecting alone, and drug dealing and weekly OPS/SCS use were associated with decreased odds.

Our study is in line with previous studies demonstrating high rates of injecting alone [13, 17, 21, 24, 43, 47]. This is significant in the context of an ongoing and escalating overdose crisis. While there is limited research on the practice of injecting drugs alone, past work suggests that stigma and shame surrounding drug use may motivate PWID to inject alone, despite the known risks [1, 46].

One common public health strategy for preventing overdose has been to focus on individual behaviour change through public messaging, including advising PWUD to never use drugs alone. Such campaigns have been shown to be limited in their effectiveness [23] and, during the COVID-19 pandemic, were complicated by narratives that advised PWUD to practise social and physical distancing [41]. While a range of overdose prevention programs based in housing and indoor settings (e.g. peer-led naloxone distribution, peer-witnessed consumption rooms, housing-based overdose prevention/supervised consumption sites) have been implemented in some areas [1, 3, 32], persistent high rates of overdose death among those using drugs alone and indoors signify a broader need for such programs and for the implementation and evaluation of novel public health interventions, such as a widely accessible safer regulated supply of drugs [9], [28]. Further, given the multitude of reasons for using drugs alone, novel overdose prevention initiatives that are tailored for those who use alone are required [3]. One such initiative that has been implemented in BC, with expansion planned for use in supportive housing settings [19], is the mobile phone overdose prevention LifeGuard App [27], though no known research has yet been conducted on its impact.

Our study found that injecting alone was strongly associated with DTES residence and binge drug use. Despite being a hub for harm reduction services, residents in the DTES experience multiple structural vulnerabilities (e.g. systemic marginalization and criminalization) that produce social isolation and loneliness and may lead to riskier drug use behaviours [1, 11, 22]. In our analysis, males were more likely to inject alone than females. This is consistent with previous studies [13, 17] and overdose surveillance data [5]. We also found that experiencing violence was associated with higher likelihood of injecting alone. This is a novel finding, although previous studies have shown that accessing harm reduction services and using drugs in public spaces or in the presence of others may present the risk of violence (often experienced differently for men and women), leading PWUD to use drugs alone to control their safety, despite increased overdose risk [8, 10, 18]. Such findings necessitate the tailoring of gender-specific and violence- and trauma-informed overdose prevention measures.

Factors that were negatively associated with injecting alone included being in a relationship and Indigenous ancestry. A partner may be viewed as a resource who can be present and attend to an overdose [35] and provide social protection and care [40]. While Indigenous people experience a disproportionate burden of substance use harms due to racism and colonization, Indigenous PWID may have relational ties and social supports that mediate drug use risks, including injecting alone [15, 36, 45].

In a sub-analysis, we found that older age and employment were associated with higher odds of always injecting alone. Previous research has shown that employment, especially when precarious, can have a destabilizing effect for PWID [38], and further evidence suggests an elevated overdose risk among those working in specific forms of work, such as construction and other service and trade industries [14, 42]. However, this unique finding and the characteristics that shape injecting alone at varying frequencies warrant further investigation. The finding of a negative association between OPS/SCS use and injecting alone adds to the existing literature on OPS/SCS by suggesting a potential positive impact on this particular overdose risk behaviour.

Our findings indicate that an expansion of urban overdose prevention programs that are based in housing and indoor settings is needed [2, 35]. However, overdose deaths that occur alone and indoors take place in all regions of BC [29], including in small and mid-sized communities [7], necessitating overdose prevention measures in diverse settings, including private market housing and in suburban, rural, and remote areas [4]. Future research should examine in which housing settings those who use alone are most at risk to inform targeted and equitable overdose prevention initiatives.

This study has limitations which should be noted. First, VIDUS and ACCESS participants are not randomly selected, and therefore, our findings may not be generalizable to PWID in our study setting or others. Second, the data were collected via self-report and may be subject to reporting biases. However, self-reported measures among PWUD have been found to be reliable [31].

## Conclusion

To conclude, our study found that injecting alone was common among our sample of PWID. These findings underscore the need for the implementation and evaluation of novel public health interventions that reduce the risk of overdose for those who inject alone, including those that are gender-specific, culturally appropriate, and violence- and trauma-informed. Further, social and cultural factors that are protective against injecting alone should be explored further for their potential relevance in overdose prevention strategy.
